# Incidence of Recurrent Major Bleeding and its Impact on Survival in LVAD Patients: A Multistate Analysis

**DOI:** 10.1055/a-2976-7540

**Published:** 2026-10-21

**Authors:** Jurjen F. Krommenhoek, Eva Janssen, Paul L. den Exter, Sabine F.B. van der Horst, Saskia L.M.A. Beeres, Jesper Hjortnaes, Meindert Palmen, Frederikus A. Klok, Nienke van Rein, Marta Fiocco, Wouter Jukema, Laurens F. Tops

**Affiliations:** 1 Department of Thrombosis and HaemostasisLeiden University Medical CenterLeidenNetherlands; 2 Department of CardiologyLeiden University Medical CenterLeidenNetherlands; 3 Department of Cardiothoracic SurgeryLeiden University Medical CenterLeidenNetherlands; 4 Department of Clinical EpidemiologyLeiden University Medical CenterLeidenNetherlands; 5 Department of Clinical Pharmacology and ToxicologyLeiden University Medical CenterLeidenNetherlands; 6 Department of Biomedical Data ScienceLeiden University Medical CenterLeidenNetherlands; 7 4496 Mathematical Institute, Leiden UniversityLeidenNetherlands; 8 Princess Máxima Center for Paediatric OncologyUtrechtNetherlands

**Keywords:** acquired coagulation disorders, intravascular devices, ischemic heart disease

## Abstract

**Introduction** Major bleeding frequently complicates the clinical course of left ventricular assist device (LVAD) therapy. However, data on recurrent major bleeding in destination therapy (DT) patients, as well as its impact on survival, are limited.

**Methods** Single-center cohort of 122 predominantly DT LVAD between 2010 and 2024. A multistate model was estimated to evaluate first and recurrent major bleeding, and all-cause mortality. Patients who died within 14 days following implantation were excluded from the analysis.

**Results** For the final analysis 116 patients (90 HeartWare HVAD; 26 HeartMate 3) were included; median age at implantation was 64 years (IQR 59–69 years); 80% were male and 56% had ischemic heart failure. During a follow-up period totaling 300 patient-years, 155 major bleeding episodes occurred in 60 patients. Half of the patients who experienced a first major bleeding ( *n* = 60) went on to develop a subsequent recurrence ( *n* = 30). Recurrence often occurred within a short interval after the initial major bleeding. Among these recurrences, 43% occurred at the same site as the initial major bleeding.

**Conclusion** Following an initial major bleeding, LVAD patients frequently experienced either recurrent major bleeding or death within a short interval. The overall incidence of (recurrent) major bleeding was high. These findings highlight the burden of recurrent bleeding events and their impact on DT LVAD patients.

## Introduction

Major bleeding is a serious adverse event frequently complicating the course of left ventricular assist device (LVAD) treatment. 1 Over the past two decades, LVADs have become an established treatment for patients with advanced heart failure (HF) either as a bridge to transplant (BTT) or as destination therapy (DT). 2 3 While LVADs were historically implanted primarily as BTT, the majority of implantations are now performed as DT, 4 5 shifting the clinical focus to the management of long-term treatment and recurrent adverse events. In LVAD patients, bleeding risk is not only increased by anticoagulation itself, but also by LVAD-related factors such as acquired von Willebrand syndrome 6 7 and angiodysplasia. 8 9 Importantly, these factors persist during long-term LVAD support in DT patients, increasing the risk of recurrent bleeding over time.

Data on the incidence of recurrent major bleeding and its impact on outcomes are limited. While most studies report only incidence rates—failing to capture the timing of recurrence—others focus solely on gastrointestinal bleeding (GIB), 10 11 12 13 thereby excluding intracranial hemorrhage (ICH), a particularly severe form of major bleeding with high comorbidity and case fatality rate. Furthermore, many studies examined major bleeding in mixed cohorts, including BTT or bridge-to-decision (BTD) patients, who are typically younger and have fewer comorbidities compared with DT patients. Therefore, there is a lack of data regarding recurrent major bleeding, including ICH, in a DT population.

We report the results of a cohort study of predominantly DT LVAD patients. A multistate model was estimated to evaluate major bleeding—both first and recurrent events—the timing of these recurrences, and survival.

## Methods

### Cohort Description and Setting

This cohort study included patients who received a HeartWare HVAD (Medtronic, Minneapolis, MN, USA) or HeartMate 3 (HM3, Abbott, North Chicago, IL, USA) at the Leiden University Medical Center (LUMC) between January 1, 2010 and August 1, 2024. Due to the discontinuation of the HVAD, the last HVAD implantation was performed in April 2021. Thereafter, only HM3 devices were implanted. At our center, all patients were treated with a combination of vitamin K antagonists (VKAs) and aspirin or clopidogrel until November 2023. After publication of the ARIES trial, patients implanted with HM3 devices received VKA monotherapy, whereas those with HVADs continued on combined VKA and antiplatelet therapy. 14 Following a major bleeding event, oral anticoagulation was typically withheld until the bleeding resolved. Hemostatic interventions, including invasive procedures and the administration of prohemostatic agents, were used based on the etiology and severity of the bleeding. Afterwards, bridging with low-molecular-weight heparin was considered until a therapeutic INR was reached, depending on the hemostatic profile of the patient.

Patients were over the age of 18, had advanced HF, and were ineligible for heart transplantation at the time of LVAD implantation. Patients who died within 14 days post-implantation were excluded ( Fig. 1 ). After reviewing the study protocol, the local medical ethics committee determined that official approval was not required under Dutch law because of the retrospective study design.

**Fig. 1:**
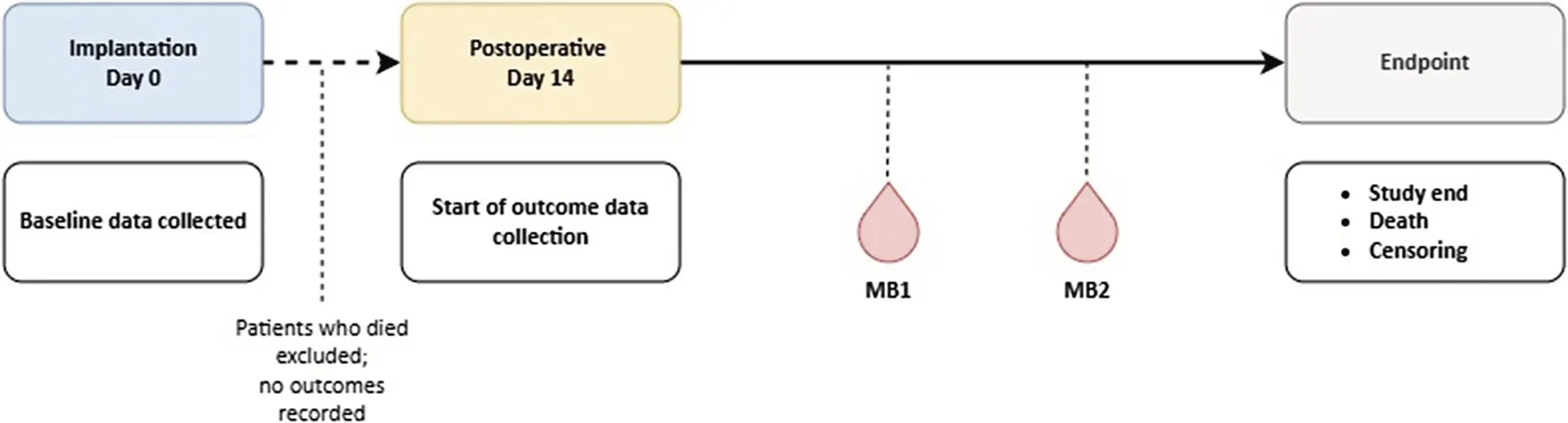
Overview of the study timeline. Patients were followed from left ventricular assist device (LVAD) implantation (day 0) through a 14-day blanking period, after which follow-up for outcomes began. Patients who died during the blanking period were excluded from the analysis. Major bleeding events (MB1, MB2) could occur at any point during follow-up. Follow-up continued until death, censoring, or the end of the study period.

### Follow-up and Outcomes

Follow-up data were collected from 2 weeks after LVAD implantation until either death or the end of the study period (August 1, 2024; Fig. 1 ). Data were collected directly from electronic health records (EHR) by our researchers to generate a detailed profile, including baseline demographics, comorbidities, surgery-related information, outcomes, duration of care, and results of laboratory and radiological tests. Our primary outcome, major bleeding (both first and recurrent), was examined through a review of EHR documentation, including clinical notes, discharge letters, blood transfusion records, and results from laboratory testing and imaging. Patients were followed up until their last recorded contact with our department or death. Patients were censored if they underwent explantation for heart transplantation or were transferred to the care of another hospital.

Major bleeding was defined according to the Mechanical Circulatory Support Academic Research Consortium (MCS-ARC) as an MCS-ARC bleeding type 3–5. 15 Additionally, MCS-ARC neurologic events type 1B (symptomatic intracerebral hemorrhage), 1C (symptomatic sub-arachnoid hemorrhage), and 1F (symptomatic sub-dural hemorrhage) were categorized as major bleeding. 15 Major bleeding episodes that occurred within a 14-day period at the same site were categorized as an ongoing episode and not as a recurrence. A sample of major bleeding episodes was cross-examined by a second researcher, and any discrepancies were resolved by a third reviewer.

### Statistical Analyses

Continuous variables were reported as mean and standard deviation if normally distributed. Otherwise, they were reported as median including interquartile ranges. The median follow-up time was estimated using the reverse Kaplan-Meier methodology and expressed as the median, minimum, and maximum durations. 16

### Incidence Rates

The incidence rates were calculated as the number of new events divided by the total patient-time at risk, expressed as events per patient-year. Confidence intervals (CIs) for the incidence rate were estimated assuming a Poisson distribution for the count of events. A normal approximation was used to calculate the 95% CI.

### Multistate Model

A multistate model was estimated to study progression of major bleeding and death over time. 17 Fig. 2 shows a schematic overview of the model, which includes four states and five possible transitions. A first major bleeding (MB1) and a second major bleeding (MB2) were considered intermediate states from which patients could transition to other states.

**Fig. 2:**
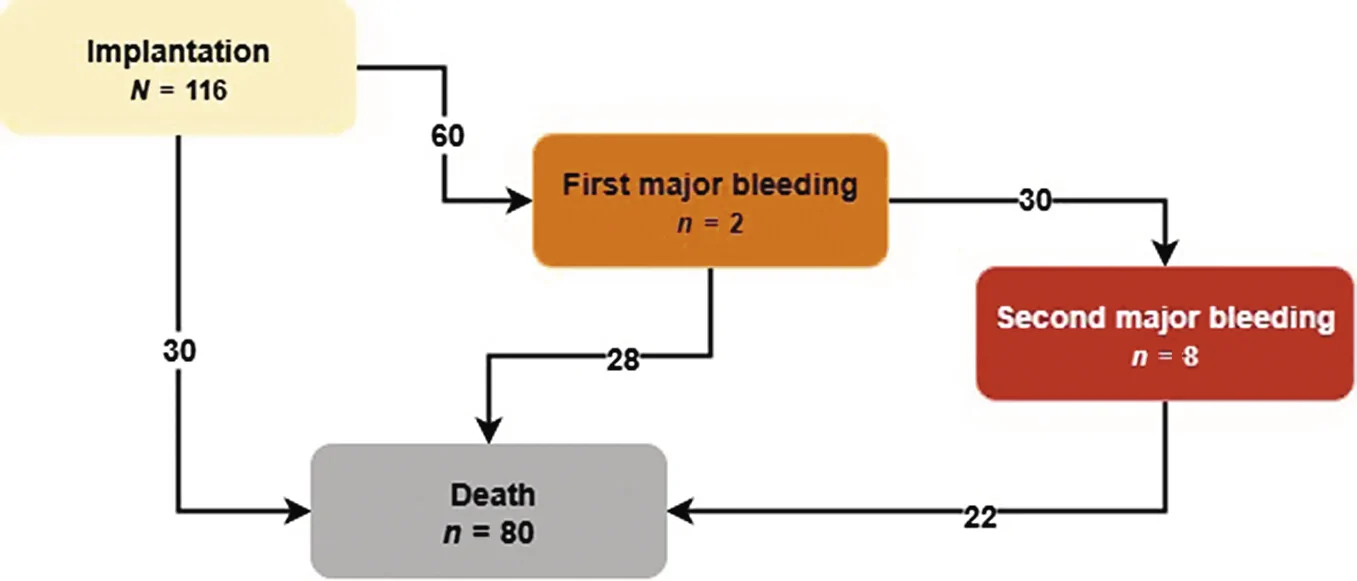
Schematic overview of the multistate model. The numbers refer to the number of patients transitioning between states. For each state, the number of patients who remain in that state and do not experience an additional event (either major bleeding or death) is shown. This model allows for five distinct transitions: Transition 1: implantation to first major bleeding; Transition 2: implantation to death; Transition 3: first major bleeding to second major bleeding; Transition 4: first major bleeding to death; Transition 5: second major bleeding to death.

The model produced two main outputs. First, the effect of risk factors for each transition was estimated by using a Cox regression model and expressed as hazard ratios (HRs) with 95% CIs. Events which occurred on the same day (i.e., bleeding and death) were offset by a small number to the death date (0.1). The number of prognostic variables was chosen based on the number of events for each transition and their clinical relevance. Due to the structure of the model, we were limited to variables available at baseline. The variables included age, sex, history of bleeding, and INTERMACS (Interagency Registry for Mechanically Assisted Circulatory Support) profile, which is a standardized 7-level classification system reflecting severity in advanced heart failure. 18 Age was treated as a continuous variable based on the age at the time of implantation. For INTERMACS profile, patients were grouped into two categories: profile 1 and 2 and profile 3 or higher. History of bleeding was defined according to the International Society on Thrombosis and Haemostasis (ISTH) definition as a documented, previous episode of either clinically relevant non-major bleeding or major bleeding. 19 The transition-specific HRs were estimated for three transitions of interest: from implantation to first major bleeding, from implantation to death, and from first to second major bleeding. Second, the state occupation probability was estimated. This probability represents the likelihood of a patient being in a particular state at a specific point in time. Occupation probability plots were generated for the entire cohort, as well as for select prognostic factors and starting states. Cumulative hazards for transition 1 (from implantation to first major bleeding) and transition 3 (from first major bleeding to second major bleeding) were also estimated. All analyses were performed using R Statistical Software (v4.1.2; R Core Team 2023). The multistate analysis was performed using the “mstate” library. 20

## Results

### Cohort Description

Of the 122 patients who received an LVAD, 6 died within the first 2 weeks, leaving a total of 116 included patients ( Table 1 ). Of these, two patients were censored during follow-up (one heart transplant, one transfer of care). Most of the patients received an HVAD ( *n* = 90, 77%), the remaining an HM3 ( *n* = 26, 23%). Median age at implantation was 64 years (Q1–Q3, 59–69). Most patients were male ( *n* = 93, 80%), had an ischemic etiology of heart failure ( *n* = 65, 56%), and received an implant with the DT device strategy ( *n* = 110, 96%). The most common INTERMACS profile was 3 ( *n* = 57, 49%). The median follow-up duration estimated using the reverse Kaplan-Meier method was 2137 days (5.9 years), with a minimum follow-up of 15 days and a maximum follow-up of 4,939 days (13.5 years).

**Table 1:** Baseline characteristics.

Characteristic	*N* = 116
Age (y), median (IQR)	63.9 (58.6–68.9)
Male, *n* (%)	93 (80.2%)
BMI, median (IQR)	25.3 (22–28.4)
Ischemic heart failure, *n* (%)	65 (56.0%)
Device strategy, *n* (%)	
Bridge to decision/transplant	6 (5.2%)
Destination therapy	110 (94.8%)
HVAD, *n* (%)	90 (77.0%)
HM3, *n* (%)	26 (23.0%)
INTERMACS profile, *n* (%)	
1	6 (5.2%)
2	18 (15.5%)
3	49 (42.2%)
4	29 (25%)
5	14 (12.1%)
Pulmonary artery pressure in mm Hg, mean (SD)	30.2 (±10)
Pulmonary capillary wedge pressure in mm Hg, mean (SD)	22.5 (±8.6)
Cardiac index, mean (SD)	1.91 (±0.42)
Hypertension, *n* (%)	36 (31%)
History of cancer, *n* (%)	25 (21.6%)
Diabetes, *n* (%)	30 (26.0%)
Chronic kidney disease, *n* (%)	55 (47.4%)
History of bleeding, *n* (%)	26 (21.6%)
Major bleeding ^a^	15 (12.9%)
Prior ischemic stroke, *n* (%)	12 (10.3%)
Cardiac arrythmia, *n* (%)	84 (72.4%)
Atrial fibrillation	64 (55.2%)
Ventricular arrythmia	48 (41.3%)
ICD implantation, ^b^ *n* (%)	78 (67.2%)
Mechanical heart valve, *n* (%)	5 (4.3%)

Abbreviations: HM3, HeartMate 3; HVAD, Heartware HVAD; ICD, implantable cardiac defibrillator; INTERMACS, Interagency Registry for Mechanically Assisted Circulatory Support.

Note: ^a^ Refers to major bleeding according to the International Society on Thrombosis and Haemostasis definition.

^b^ Combination of ICD and cardiac resynchronization therapy devices (CRT-D).

### Major Bleeding

A total of 155 major bleeding episodes were observed ( Table 2 ). The most common were GIB ( *n* = 92, 49%), followed by ICH ( *n* = 18, 12%) and intrathoracic bleeding ( *n* = 15, 9.7%). The incidence rates of major bleeding and GIB were 0.51 (95% CI: 0.38–0.78) and 0.31 (95% CI: 0.19–0.42) per patient-year, respectively. More than half of the patients ( *n* = 60, 52%) experienced a major bleeding, of whom 50% had recurrent episodes ( Table 2 ). Additionally, 10.3% ( *n* = 16) experienced three or more major bleeding episodes. Of the 30 patients who experienced a recurrent major bleeding, 43% ( *n* = 13) had recurrence at the same anatomical site. Of the 16 patients who experienced a third major bleeding, 9 (56%) had a same site recurrence, of which 8 were recurrent GIB.

**Table 2:** Major bleeding during follow-up.

Bleeding episodes	*n* = 155
Bleeding site	
Gastrointestinal	92 (59.4%)
Intrathoracic ^a^	15 (9.7%)
Intracerebral	18 (11.6%)
Epistaxis	9 (5.8%)
Hematoma	9 (5.8%)
Other ^b^	12 (7.7%)
**Number of major bleeding events among patients**	***N* = 116 **
≥1 major bleeding ^c^	60 (51.7%)
≥2 major bleeding	30 (25.8%)
≥3 major bleeding	16 (10.3%)
≥4 major bleeding	9 (7.7%)
Recurrent bleeding at the same site	
First to second major bleeding	13 (43%) *N* = 30 patients
Second to third major bleeding	9 (56%) ^d^ *N* = 16 patients

Note: Data are expressed as *n* (%).

^a^ Intrathoracic is a combined category including mediastinal, intrathoracic, pericardial, and other types of device-related bleeding.

^b^ Includes abdominal and retroperitoneal bleeding.

^c^ Categories are cumulative and not mutually exclusive (e.g., patients with ≥2 major bleedings are also included in the ≥1 category).

^d^ 8/9 gastrointestinal bleeding.

### Transition-specific HRs

A history of any bleeding was associated with a non-significant increased risk of a first major bleeding (HR = 1.46, 95% CI: 0.82–2.62), as shown in Table 3 . No association was found between age, sex, or INTERMACS profile (1–2) and the risk of a first major bleeding. Regarding the transition from implantation to death, an INTERMACS profile 1 or 2 and age were associated with an increased risk of mortality (HR = 4.02, 95% CI: 1.73–9.32 and HR = 1.07, 95% CI 0.98–1.12, respectively). For the transition from the first to the second major bleeding, no association was identified between age, sex, or history of bleeding and the risk of recurrence.

**Table 3:** Estimated hazard ratio (HR) along with 95% confidence interval (CI) from a Cox proportional hazard regression for different transitions in the multistate model.

Variable	Implantation → first major bleeding HR (95% CI)	Implantation → death HR (95% CI)	First major bleeding → second major bleeding HR (95% CI)
Age ^a^	1.01 (0.98–1.04)	1.07 (1.01–1.12)	1.02 (0.98–1.07)
Sex (male)	1.00 (0.52–1.93)	0.92 (0.37–2.3)	0.70 (0.26–1.85)
INTERMACS profile ^b^	1.09 (0.53–2.26)	4.02 (1.73–9.32)	
History of bleeding ^c^	1.46 (0.82–2.62)		0.89 (0.39–2.03)

Abbreviation: INTERMACS, Interagency Registry for Mechanically Assisted Circulatory Support.

Note: ^a^ Age was expressed as continuous variables in years at implantation.

^b^ INTERMACS profile (1–2).

^c^ Clinically relevant non-major bleeding and major bleeding according to the International Society on Thrombosis and Haemostasis definition.

### Transition Risk

The cumulative hazard of a first major bleeding increased most sharply within the first 100 days following LVAD implantation before stabilizing ( Fig. 3 ). In contrast, the cumulative hazard for recurrent major bleeding (transition 3: first to second major bleeding) increased more steadily and at a higher rate over time.

**Fig. 3:**
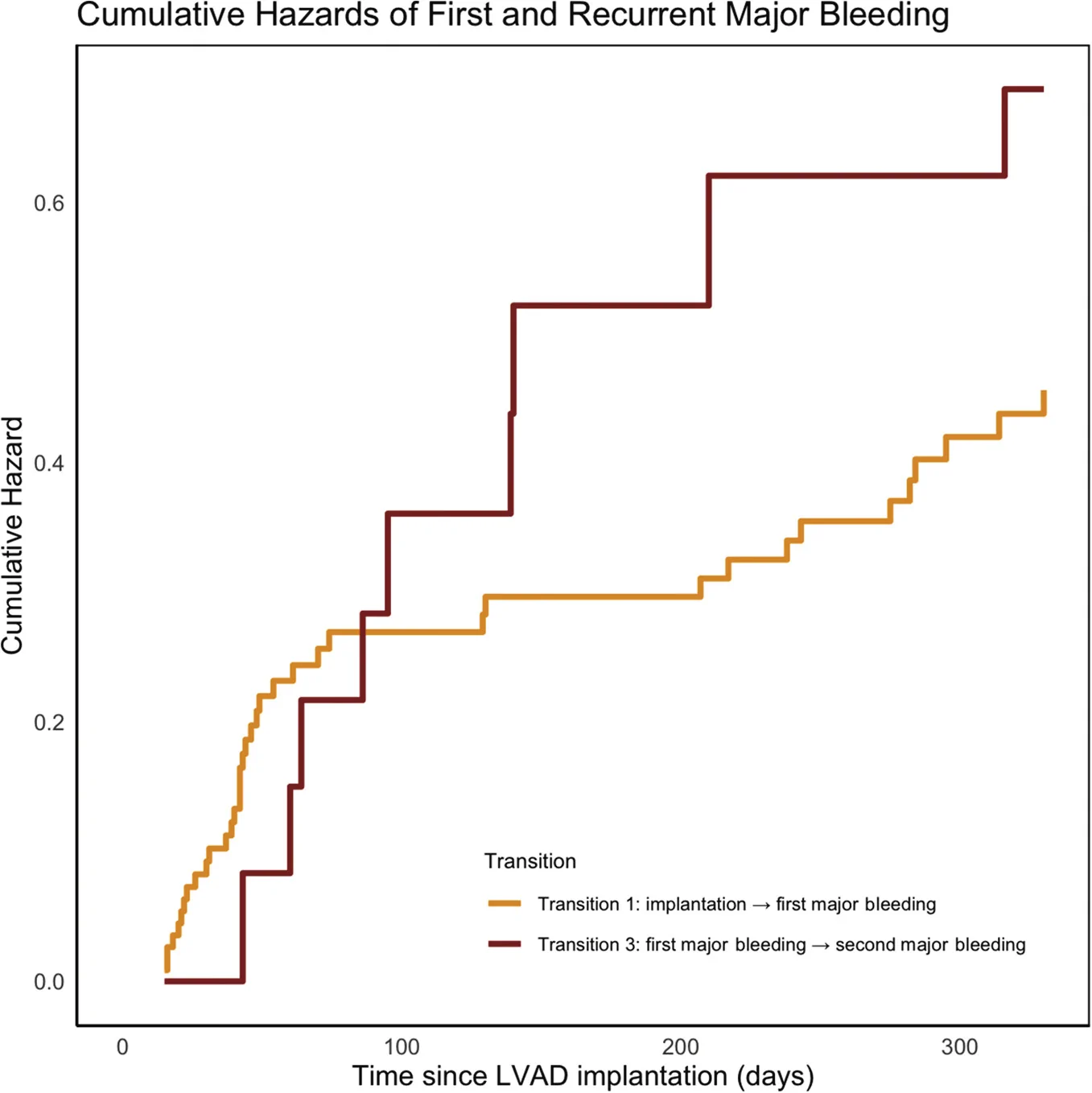
Cumulative hazards for transition 1 and transition 3 during the first year following left ventricular assist device (LVAD) implantation. Cumulative hazards for the transition from LVAD implantation to first major bleeding (transition 1) and from first to second major bleeding (transition 3), plotted against time since LVAD implantation, during the first year of follow-up. Cumulative hazard for transition 3 is shown only for patients who reached the first major bleeding state and is plotted using the same implantation based time origin as transition 1.

### State Occupation Probability

Fig. 4 shows the probability of a patient being in each state at a particular time after LVAD implantation. The rates of major bleeding and death were highest during the first 6 months. During this period, approximately 30% of patients experienced at least one event (major bleeding or death), rising to 50% at 1 year. Thereafter, the event rate decreased and remained relatively stable during the remainder of follow-up.

**Fig. 4:**
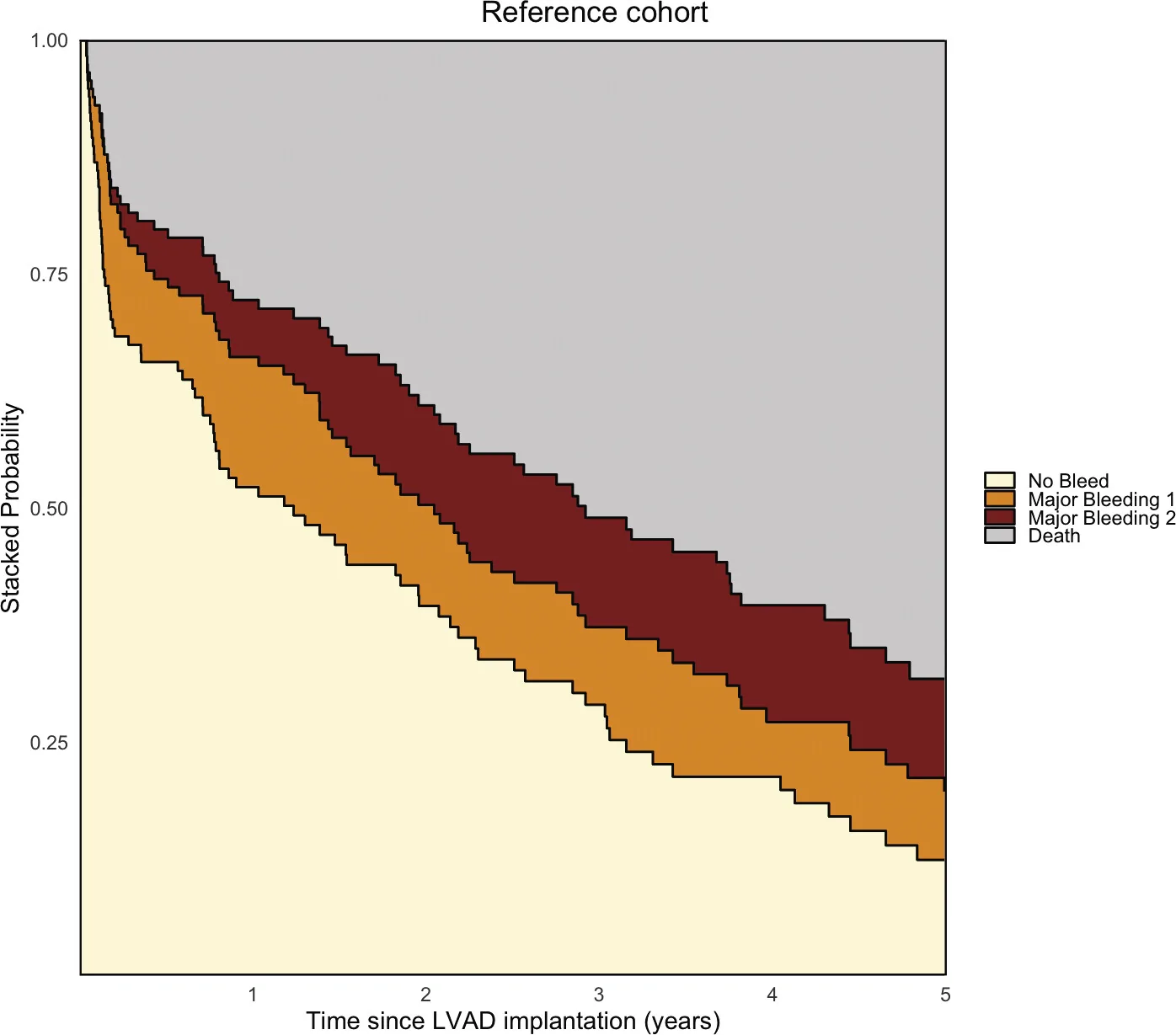
State occupation probability plot. State occupation probability plot for the overall cohort, showing the probability of a patient being in each state (implantation without a major bleeding event, first major bleeding [MB1], second major bleeding [MB2], or death) at a given time since left ventricular assist device (LVAD) implantation.

## Discussion

In this study, we applied a multistate model to evaluate major bleeding—both first and recurrent—and survival in a LVAD-DT cohort. We found that patients who experienced an initial major bleeding had a high probability of a subsequent major bleeding or death within a short interval. Additionally, a substantial proportion of patients had an adverse event (either major bleeding or death) within the first 6 months following implantation, after which the event rate appeared to stabilize. Among the prognostic factors we examined, INTERMACS profile 1 or 2 was associated with increased post-implant mortality, and there was a trend suggesting that a history of bleeding prior to implantation increased the risk of a first major bleeding.

Our primary finding was that patients with an initial major bleeding had a high probability of recurrence, often within a short interval. Prior studies have also reported high recurrence rates of between 40% and 80%, but few have examined the timing of these recurrences. 10 11 For example, one observational study reported 87 major bleeding episodes among 43 patients—most of which were GIB—yet did not provide data on the temporal relationship between episodes. 10 Similarly, a recent INTERMACS analysis reported that after a first episode of GIB there was a 4-fold risk of recurrence. 5 However, the timing of these subsequent episodes was not evaluated, and an increased risk does not necessarily imply a shorter interval between the initial and subsequent episodes. While GIB has been identified as a major driver of recurrence, only 43% of recurrent major bleeds in our study occurred at the same anatomical site. Additionally, we observed that death frequently followed a major bleeding episode, though our study was not designed to establish causality, as such an association may be influenced by confounding factors unrelated to bleeding.

We also observed a pattern in which the incidence of major bleeding and death was highest during the 6 months following LVAD implantation, after which the incidence rates appeared to stabilize. This pattern has also been seen in previous studies. For example, in a multistate analysis of the MOMENTUM-3 trial, the risk of GIB and death was highest during the first 3 months following implantation. 21 Similarly, in the INTERMACS 2024 annual report, the incidence of early bleeding (<90 days) was nearly six times higher than that of late bleeding (>90 days). 5 This time trend was also observed among patients who experienced a first major bleeding, with a significant proportion experiencing a subsequent clinical event (either recurrent major bleeding or death) within the following 6 months.

In comparison with previous literature, we report a relatively high incidence of major bleeding. 4 5 10 11 We offer two potential explanations for this discrepancy. First, in our study, the majority of LVADs were implanted as DT. In general, DT patients are older and have more comorbidities. This may have resulted in higher rates of adverse events (including major bleeding) compared with mixed strategy cohorts. 4 5 10 Second, unlike the MCS-ARC definition, our definition of major bleeding included ICH. It was decided to include ICH in our definition because of its high case fatality and morbidity rates, 22 as well as its inclusion in the definitions of major bleeding outside the LVAD field. 19

As expected, an INTERMACS profile of 1 or 2 was associated with a higher risk of post-implantation mortality, likely due to more severe organ failure and complications related to the treatment of cardiogenic shock. We also observed a statistically non-significant increased risk of a first major bleeding in patients with a history of bleeding. This finding aligns with previous research, as a history of bleeding has been recognized as a predictor of GIB in both the Michigan and Utah LVAD-specific GIB risk scores. 12 23 However, it is important to emphasize that this association does not necessarily imply an underlying causal relationship, since prediction should not be confused with causality. In addition, the limited data available in our study precludes us from a more in-depth analysis on the recurrence pattern of major bleeding. Nonetheless, the results of our study emphasize the high burden of recurrent bleeding events in LVAD patients.

Given the high recurrence rates of major bleeding observed in our study and others, 10 12 21 along with the short interval to subsequent major bleeding seen in our cohort, there is a clear need to focus on secondary preventive measures. There are currently several strategies. One of the most promising approaches is reducing the intensity of anticoagulation. This has already been initiated with the previously mentioned shift from dual therapy (VKA plus antiplatelets) to monotherapy (VKA) in HM3 patients. 14 Lower device thrombosis rates observed with newer models such as the HM3 have promoted the adoption of less intensive anticoagulation regimens. Current research is investigating further reductions in anticoagulation intensity, primarily in the form of pilot trials in HM3 cohorts. These trials include lowering the target INR range to 1.5–1.9 and evaluating changes in anticoagulant class, such as switching from VKAs to direct oral anticoagulants (DOACs) or antiplatelet monotherapy. 24 25 26 While these results are promising, confirmation through large-scale randomized controlled trials is needed to establish safety and efficacy. A potential limitation of these strategies, should they prove viable, is that they are currently restricted to HM3 patients. Other secondary prevention strategies mostly target GIB, and include the use of octreotide, 27 28 proton pump inhibitors, angiotensin-converting enzyme inhibitors, 29 and vascular endothelial growth factor inhibition. 30 The effectiveness of these interventions remains uncertain, as they are supported mainly by small-scale observational studies or case series.

Multistate modeling allowed us to evaluate (recurrent) major bleeding dynamically and to study the effects of specific characteristics on the course of disease progression. Our cohort was relatively large, including both HVAD and HM3 devices, and was composed of an almost homogeneous DT population. Compared with larger registries, our study offers more complete follow-up data spanning nearly 14 years. However, our study has limitations that need to be discussed. Our results are from a single center, which may not be generalizable to other types of populations (e.g., BTT) and partially reflect local practice and LVAD referral patterns. The censoring period may have led to the exclusion of some bleeding episodes that occurred within 14 days but originated from different sources of the same type, for example, two distinct types of GIB. Furthermore, most of our patients (77%) were HVAD recipients, the distribution of which was discontinued in 2021. There is conflicting evidence as to how similar (major) bleeding rates are between HVAD and HM3 recipients. Most studies report a reduced incidence of bleeding in HM3 patients, although to varying degrees. 31 32 33 34 35 36 These studies were conducted before the shift toward less intensive anticoagulation in HM3 patients, so the difference may currently be more pronounced. Nevertheless, the incidence of major bleeding remains substantial, and many recipients of older devices, including HVADs, will continue requiring care for years to come.

Another limitation is that thrombosis and related treatments may have influenced some of the associations we observed—for example, adjustments in anticoagulation regimens or administration of thrombolysis following device thrombosis. The latter was investigated in our cohort: among 14 patients treated for pump thrombosis, the 30-day major bleeding rate was 21%. Therefore, we believe the impact of this factor on overall outcomes is likely limited. 37 Time in therapeutic range (TTR) is also a determinant of bleeding, but as our data were time fixed at baseline, we could not incorporate TTR into our multistate model or evaluate its association with bleeding risk in our cohort. Finally, our sample size was limited. As patients transition through the states, the number of patients decreases sharply, reducing the precision of risk estimates. To address this, we restricted our model to four states and five corresponding transitions.

## Conclusion

Following an initial major bleeding, LVAD patients frequently experienced either recurrent major bleeding or death within a short interval. The overall incidence of (recurrent) major bleeding was high. These findings highlight the burden of recurrent bleeding events and their impact on DT LVAD patients.

## Data Availability

The data underlying this study are not publicly available due to institutional and privacy restrictions related to patient information. Data may be made available from the corresponding author upon reasonable request, subject to approval from the relevant institutional review board.
